# Cardiac Tamponade in Patients With Breast Cancer: A Systematic Review

**DOI:** 10.7759/cureus.33123

**Published:** 2022-12-30

**Authors:** Nosakhare Ilerhunmwuwa, Ephrem Sedeta, Mustafa Wasifuddin, Narek Hakobyan, Henry O Aiwuyo, Jamal C Perry, Ifeanyi Uche, Kennedy Okhawere, Beatrice E Torere, Erdinc Burak, Heravi Omid, Jen Chin Wang

**Affiliations:** 1 Internal Medicine, Brookdale University Hospital Medical Center, Brooklyn, USA; 2 Medicine, Brookdale University Hospital Medical Center, Brooklyn, USA; 3 Urology, Icahn School of Medicine at Mount Sinai, New York City, USA; 4 Internal Medicine, North Mississippi Medical Center, Tupelo, USA; 5 Hematology and Oncology, State University of New York (SUNY) Downstate Health Sciences University, New York City, USA; 6 Hematology and Oncology, Brookdale Hospital Medical Center/One Brooklyn Health, Brooklyn, USA; 7 Hematology/Oncology, Brookdale University Hospital Medical Center, Brooklyn, USA

**Keywords:** pericardial effusion, prisma, breast cancer, systematic review, cardiac tamponade

## Abstract

Cardiac tamponade is a rare presentation in breast cancer and may be associated with poor prognosis. In this article, we reviewed the characteristics and survival outcomes of patients with breast cancer who developed cardiac tamponade. Three databases (PubMed, EMBASE and SCOPUS) were searched for relevant articles published from 1978 to 2022 and 16 articles were identified comprising 64 cases. The median age of the cases was 52 years. Cardiac tamponade was diagnosed with echocardiogram or computerized tomography of the chest or both in 91.9%, 1.6% and 6.5% of the cases, respectively. Cytology of the pericardial fluid was done in 90.5% of the cases while biopsy in addition to cytology was done in 9.5% of cases. Tamponade was proven to be malignant in 97.4% of the cases. The initial treatment for tamponade was pericardiocentesis. Adjunct therapies ranged from the insertion of a pericardial window, pericardiectomy, radiotherapy and chemotherapy. The median time from the first treatment of breast cancer to the onset of tamponade was 24 months while the median survival following diagnosis of tamponade was 13 months. There was no significant correlation (spearman rank-sum correlation coefficient= 0.35, p = 0.165) between time to tamponade (interval time from the first diagnosis of breast cancer and the onset of cardiac tamponade) and survival. Cardiac tamponade may adversely affect survival in patients with breast cancer. Early diagnosis with echocardiogram and cytology may guide management and expectations. Further observational studies are needed to determine the predictors of cardiac tamponade and optimal treatment in patients with breast cancer.

## Introduction and background

Cardiac tamponade is a rare presentation in breast cancer with a reported prevalence of less than 1% [1-4]. It results from acute or chronic pericardial effusion which impairs diastolic filling and venous return to the heart. This ultimately reduces cardiac output, leading to cardiogenic shock and death. Cardiac tamponade in breast cancer is an emergency that requires early detection and prompt management based on available evidence.

Pericardial effusion leading to cardiac tamponade in breast cancer may be due to direct involvement or metastatic spread of the underlying malignancy to the pericardium, the effects of radiation or systemic antineoplastic therapies. The onset of cardiac tamponade may impact negatively the outcomes of breast cancer [4]. Due to the rarity of cardiac tamponade in breast cancer, studies are restricted to mostly case reports and case series with varying findings. We performed a systematic review of the available literature to explore the characteristics and survival outcomes in patients with breast cancer who develop cardiac tamponade.

## Review

Search strategy

The Preferred Reporting Item for Systematic Review and Meta-Analysis (PRISMA) guidelines were followed in this systematic review [5]. Three electronic databases (PubMed, EMBASE and SCOPUS) were comprehensively searched for articles on breast cancer and cardiac tamponade published until July 14, 2022. The search was done using the following subject headings: breast cancer, breast carcinoma, and cardiac tamponade. No specific search filters were applied.

References were uploaded to Zotero v5.0.81 (Zotero.org). This reference manager automatically identified duplicated articles which were removed. Two authors manually and carefully checked the remaining references to ensure all duplicate articles had been removed.

Study selection

The inclusion criteria for eligible studies were confirmed diagnosis of cardiac tamponade, breast cancer (primary), human subjects, and extractable data. The exclusion criteria included pericardial effusion without cardiac tamponade, co-existing primary cancers, other cancers with metastases to the breast presenting with cardiac tamponade, articles in any other language apart from English, animal subjects, non-extractable data, and post-mortem diagnosis of breast cancer and/or cardiac tamponade.

Two authors independently selected eligible articles based on the inclusion and exclusion criteria. This was done in two stages: screening the titles and abstracts of the articles for relevance and then, examination of the full texts of the relevant articles to determine those that met the inclusion criteria. Articles that did not meet these criteria were excluded. A third author resolved disagreements between the two authors involved in this process.

Data extraction

The following data were extracted from each article: first author and year of publication, country, the number of patients, study design, gender, age (years), the diagnostic tool for cardiac tamponade, laterality of breast cancer, histology of breast cancer, malignant or benign tamponade, time to tamponade, treatment for tamponade, re-accumulation of pericardial effusion, survival time (months), and vital status at the end of follow-up. Time to tamponade was defined as the interval time from the first treatment of breast cancer to cardiac tamponade, and survival time was defined as the time from the first treatment of cardiac tamponade to the date of death or last follow-up.

Quality assessment

The quality of each one of the eligible 16 articles was assessed by two authors using the tool and approach suggested by Murad et al. [6]. A third author resolved disagreements in judgment. The tool was proposed for assessing the quality and possible risk of bias of studies used in the systematic review of case reports/case series. It evaluates studies in four domains: selection, ascertainment, causality, and reporting. The domain of ascertainment and causality have two and four sub-groups respectively. This makes up a total of eight items in the tool.

For this systematic review, each domain was assigned one (1) point. For domains that have sub-groups, their assigned weighted score (1) was divided by the number of sub-groups. A zero point was assigned to an item if the answer is in negative. Three items in the causality domain (questions of other alternative causes that may explain the observation ruled out, challenge/rechallenge phenomenon, and presence of dose-response effect) were excluded due to their irrelevance to this present systematic review resulting in a final assessment tool presented in Table 5. Based on this, the following grading system was used to determine the quality: 0 - 2 (low quality), 3 (moderate quality) and 4 (good quality).

Statistical analyses

All statistical analyses were conducted using Microsoft Excel (Profession plus 2016, USA). Patient-level data were extracted from the studies, and all analyses were based on available cases. All variables were converted to a uniform scale of measurement, for example, survival time in years was converted to months. Categorical variables were presented with frequency and percentages, while continuous variables were presented with median and ranges. A spearman rank-sum correlation was used to assess the relationship between time to tamponade, and survival time after treatment of cardiac tamponade. Statistical significance was determined at p < 0.05.

Literature search results

**Figure 1:**
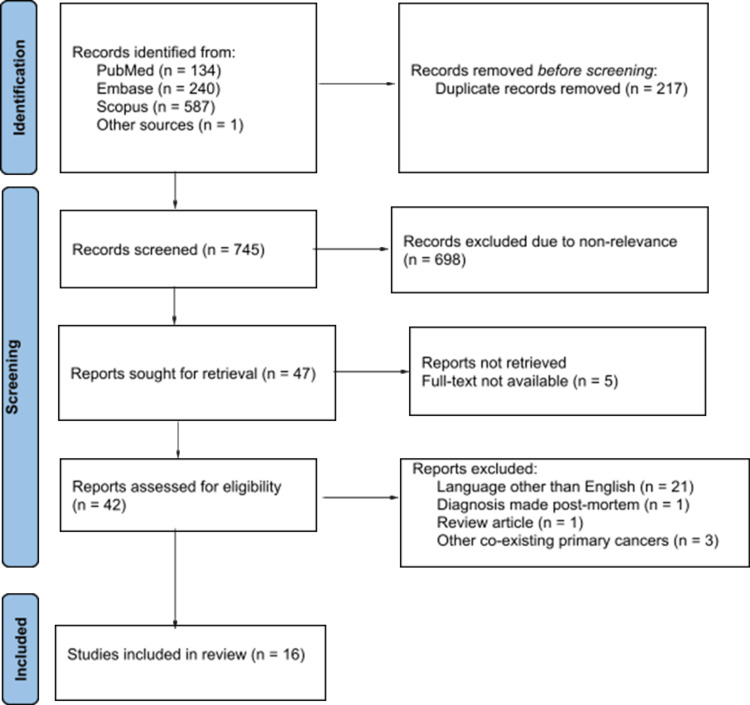
PRISMA flowchart of the identification of studies via databases

Our literature search yielded 962 articles (Figure 1) with a total of 765 articles after removing duplicates. Initial screening of the studies by title and abstract resulted in 47 articles for full-text review while a final total of 16 articles was obtained after satisfying the set-out criteria for qualitative and quantitative analysis.

Quality assessment results

The quality of the included studies and the domains in which each study was deficient in quality are shown in Table 6. Ten of the studies had good or moderate quality while the remaining 6 were rated low.

Study overview

Sixteen studies (Table 1) published from a period of 1978 and 2022, with a total of 64 patients with breast cancer and cardiac tamponade were included in this systematic review. Of these, five studies were from the United States, two from the United Kingdom, eight were from Asia, and one from Europe. Regarding the study designs, 11 were case reports, one was a case series, and four were cohort studies.

**Table 1:** Synthesized data from the selected studies NS: not specified; ECHO: echocardiogram; CT: computerized tomography; E: estrogen; P: progesterone; Her 2: human epidermal growth factor receptor 2; *: In addition, four patients had systemic chemotherapy; ↕: death was related to re-accumulation of pericardial effusion and recurrence of tamponade

Author	Study Design	Cases	Median age (years)	Age range (years)	Laterality of Breast Cancer	Histology of Breast Cancer	Immunohistochemistry of breast cancer	Prior treatment of breast cancer	Diagnosis of Tamponade	Pericardial involvement (Biopsy or Cytology)	Pathology of Pericardial Fluid	Other sites of metastases	Time to tamponade, months median (range)	Treatment of Tamponade	Recurrence of pericardial effusion	Survival time, months median (range)	Vital status
Almagro, 1982 [7]	case report	1	47	-	Left	Invasive ductal carcinoma	NS	None	ECHO	Biopsy and Cytology	Malignant	Lymph nodes	0	Pericardiocentesis, pericardiectomy	No	0.13	Dead
Almajed, 2022 [8]	case report	1	40	-	Right	Invasive ductal carcinoma	NS	None	ECHO	Cytology	Malignant	Bone	24*	Pericardiocentesis, pleuropericardial window	Yes	NS	NS
Bishiniotis, 2000 [1]	cohort	19	53	22-71	NS	NS	NS	NS	ECHO	Malignant		Lung (at least in 8 patients)	NS	Pericardiocentesis, intrapericardial chemotherapy (Thiotepa)	Yes,	10.9 (0.5 - 34.2)	Dead
Bitran, 1984 [2]	cohort	6	58	47-68	NS	NS	NS	NS	ECHO	Cytology (n=6)	Malignant	brain, bone, liver	27 (19 – 156)	Pericardiocentesis, pleuropericardial window, intrapericardial tetracycline*	No	21.5 (1 – 50)	Dead
Davis, 1978 [9]	case report	1	57	-	NS	Invasive ductal carcinoma	NS	NS	NS	Biopsy and Cytology	Malignant	NS	NS	Pericardiocentesis intrapericardial tetracycline	No	8.7	Dead
Einama, 2006 [10]	case report	1	46	-	Left	invasive ductal carcinoma	E+/P+/Her 2 -	Surgery, chemotherapy	ECHO + CT	Cytology	Malignant	Lymph node, liver	56.4	Pericardiocentesis	No	5.9	Dead
Ikeda, 2013 [11]	case report	1	55	-	Left	NS	E-/P+/Her 2 -	Surgery, radiotheraphy	ECHO + CT	Cytology	Malignant	None	28.8	Pericardiocentesis and pleuropericardial window	Yes	57	Dead
Imamura, 1989 [12]	case report	1	52	-	Left	NS	NS	Surgery, chemotheraphy	NS	Cytology	Malignant	Lymph nodes, bones and lungs	24	Pericardiocentesis with intrapericardial OK-432 instillation	No	7	Dead
Ismail, 1991 [13]	case report	1	49	-	Left	Invasive ductal carcinoma	NS	Surgery, radiotheraphy	ECHO	Cytology	Malignant	None	5	Pericardiocentesis	Yes	0.3	Dead
Konishi, 2012 [14]	case series	3	39	33-60	Left (2), bilateral (1)	Invasive ductal carcinoma	E+/P+/Her 2 -; E-/P+/Her 2; E+/P+/Her 2-	Surgery, chemotherapy, radiotherapy	ECHO , CT	Cytology (n=3)	Malignant	liver, lungs, lymph node, peritoneum	90 (94.8 - 116.4)	Pericardiocentesis with intrapericardial cisplatin	No	14 (13 – 31)	Dead
Koyama, 1999 [15]	case report	1	49	-	Left	NS	NS	Surgery, radiotherapy	ECHO	Biopsy and Cytology	Benign	None	29	Pericardial drainage, partial pericardiectomy, pericardio-venous shunt	Yes	17	NS
Lv et al, 2016 [16]	case report	1	44	-	Right	Invasive ductal carcinoma	E-/P-/Her 2 +	Chemotherapy, rsdiotherapy	CT	Cytology	Malignant	Axillary LN's only	0	Pericardiocentesis with intracavitary chemotherapy (cisplatin)	Yes	7	Dead↕
Ramakrishnan, 1988 [3]	cohort	3	57	49-69	NS	Adenocarcinoma (sub-type NR)	NS	Surgery, endocrine, radiotherapy	ECHO	Cytology (n=3)	Malignant	brain, lung, bone, intra-abdominal	28 (0 – 78)	Pericardiocentesis	No	14 (14 – 16)	Dead
Stitt, 1987 [17]	case report	1	54	-	Left	NS	NS	Surgery	ECHO	Biopsy and Cytology	Malignant	NS	NS	Pericardiocentesis, pleuropericardial window	No	12	Alive
Ucche, 2022 [18]	case report	1	55	-	Left	Invasive ductal carcinoma	E+/P-/Her 2 -	No	ECHO	Cytology	Malignant	Brain, bone	3	Pericardiocentesis, pleuropericardial window	No	12	Dead
Woll, 1987 [4]	cohort	22	48	25-65	NS	NS	NS	Endocrine, chemotherapy	ECHO	NS	NS	Yes but not specified	NS	Pericardiocentesis, pleuropericardial window (1 patient), intrapericardial chemotherapy, bleomycin (1 patient)	NS	8.8 (NS)	NS

Demographic and baseline characteristics of study participants

The demographic and clinical characteristics of the study participants are shown in Table 2. The median age of the study participants was 52 years and ranged from 22 to 71. All cases were females. The proportion of patients with left breast cancer based on 13 cases reported [7,8,10-18] was 76.9% (n=10). The histology of breast cancer was reported in 9 studies [3,7-10,13,14,16,18], and the majority were invasive ductal carcinoma 90.9% (n=10). Five studies [10,11,14,16,18] described the immunohistochemistry status of breast cancer in 7 cases; human epidermal growth factor receptor 2 (Her 2) was negative in 85.7% (n=6) of cases. Fifteen studies reported the method of diagnosis of tamponade; echocardiogram alone was used in 92% (n=57) of cases, echocardiogram with computerized tomography (CT) scan of the chest was employed in 6.5% (n=4) of cases and CT scan of the chest alone in one case.

**Table 2:** Demographic and baseline clinical characteristics of study participants ER: estrogen receptor; PR: progesterone receptor; Her 2: human epidermal growth factor 2; CT: computed tomography

Variable	
Age (years)	52 (22-71)
Gender	
Female	64 (100%)
Male	0 (0%)
Laterality of Breast Cancer (n=13)	
Left	10 (76.9%)
Right	2 (15.4%)
Bilateral	1 (7.7%)
Histology of Breast Cancer (n=11)	
Invasive Ductal Carcinoma	10 (90.9%)
Adenocarcinoma (subtype unspecified)	1 (9.1%)
Immunohistochemistry of Breast Cancer (n=7)	
ER+ / PR+ / Her 2-	3 (42.8%)
ER- / PR+ / Her 2-	2 (28.6%)
ER+ / PR- / Her 2-	1 (14.3%)
ER- / PR- / Her 2+	1 (14.3%)
Diagnosis of Tamponade (n=62)	
Echocardiogram	57 (91.9%)
CT Scan chest	1 (1.6%)
CT scan chest and Echocardiogram	4 (6.5%)

Pathologic characteristics of study participants

Pathology of cardiac tamponade was reported in 42 cases [1,2,7-18]. Cytology of the pericardial fluid was conducted in 90.5% (n=38), and biopsy in addition to cytology was done in 9.5% (n=4) of the cases (Table 3). The pericardial fluid was malignant in 97.4% (n=42) of the cases and benign in one patient. From the studies that reported the time to tamponade [2,3,7,8,10-16,18], the median time to the occurrences of cardiac tamponade after the first cancer treatment was 26 months (0-156).

**Table 3:** Pathologic characteristics of study participants

Variable	
Diagnostic pathologic tool of pericardial involvement (n=42)	
Cytology only	38 (90.5%)
Biopsy only	0 (0.0%)
Cytology and biopsy	4 (9.5%)
Pathology of pericardial effusion (n=42)	
Malignant	41 (97.6%)
Benign	1 (2.4%)

Metastasis of breast cancer to other secondary sites apart from the pericardium was reported in 11 studies [1-4,7,8,10,12,14,16,18]. The common sites were lungs, bone, peritoneum, lymph nodes, brain, and liver. There was no evidence of metastases to other extra-pericardial secondary sites in three of the included studies [11,13,15].

Intervention and outcomes

The different intervention measures used in the management of the cardiac tamponade and the clinical outcomes are shown in Table 4. The initial treatment for tamponade in all cases was pericardiocentesis [1-4,7-18]. In 42.2% of cases (n=27), pericardiocentesis was the only treatment they received, 37.5% (n=24) had additional chemotherapy (local instillation of sclerosing agent or systemic chemotherapy), 10.9% (n=7) had surgical treatment (pericardiectomy or pericardial window), and 9.4% (n=6) had all the three therapeutic approaches (pericardiocentesis, surgery, and chemotherapy). Recurrence of pericardial effusion after treatment of cardiac tamponade was assessed in 42 cases [1-3,7-18]; 21.4% (n=9) had re-accumulation of pericardial effusion while there was none in 78.6% (n=33) of the cases.

**Table 4:** Intervention and outcomes of study participants

Variable	
Time to Tamponade (months)	26 (0-156)
Treatment of Tamponade (n=64)	
Pericardiocentesis only	27 (42.2%)
Pericardiocentesis + Chemotherapy	24 (37.5%)
Pericardiocentesis + Surgery	7 (10.9%)
Pericardiocentesis + Chemotherapy + Surgery	6 (9.4%)
Recurrence of Pericardial Effusion (n=42)	
No	33 (78.6%)
Yes	9 (21.4%)
Survival (Months)	13 (0.13-57)
Vital Status (n=40)	
Alive	1 (2.5%)
Dead	39 (97.5%)

**Figure 2:**
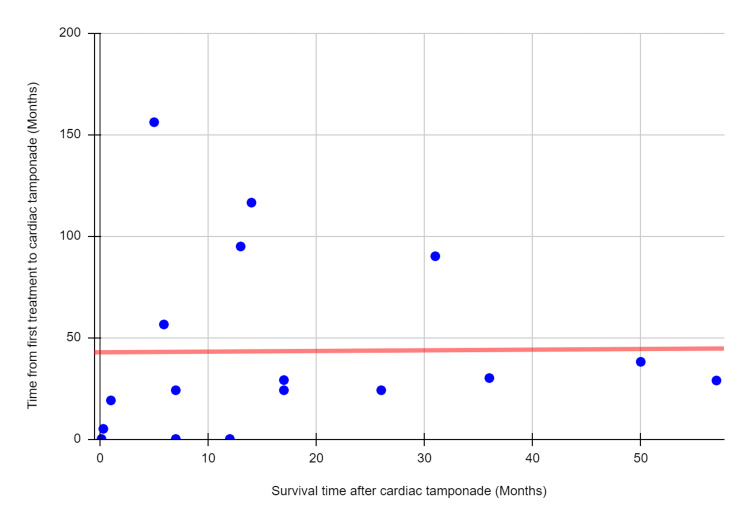
Correlation between the time from first treatment of breast cancer to the development of cardiac tamponade and survival time after treatment of cardiac tamponade

Fifteen studies reported survival time [1-4,7,9-18]. The median survival time after diagnosis of tamponade was 13 months (0.13-57) (Table 4). Thirteen studies [1-3,7,9-14,16-18] reported the vital status of the patients at the end of the follow-up. Of the 40 cases reported, 97.5% (n=39) were reported dead and none of them was related to the recurrence of pericardial effusion except in one case. As shown in Figure 2, there was no significant correlation between time to tamponade and survival time after treatment of cardiac tamponade (n = 17, spearman rank-sum correlation coefficient = 0.35, p = 0.165). Survival data were not available for 24 out of the 64 patients in this review.

Discussion

To the best of our knowledge, this is the first systematic review that examined the characteristics and survival outcomes of patients with breast cancer who develop cardiac tamponade. All the cases were females. Breast cancer is about 100 times more common in females than in males [19]. We found that cardiac tamponade was more frequently described with left breast cancer than the right breast. Breast cancer occurs more commonly on the left breast than the right and has more aggressive biologic characteristics with worse outcomes than the latter [20-23].

In this study, cardiac tamponade was more frequently associated with invasive ductal carcinoma of the breast compared to other histologic types. Invasive ductal carcinoma is the most common histological type of breast cancer [24,25]. The aggressive nature of this type of breast cancer and the anatomic proximity of the breast to the heart partly explain why it commonly metastasizes to the pericardium. For the studies that reported the immunohistochemistry status in our review, most of the cases in our review were hormone receptor-positive (either estrogen receptor [ER] or progesterone receptor [PR] or both) and Her-2 receptor negative; this should expectedly, as shown by some studies [26-28], mean a less aggressive tumor type, reduced recurrence including effusion and a relatively good prognosis. However, other studies have demonstrated that a negative ER or PR receptor status alone in breast cancer is associated with more aggressiveness, recurrence and poorer prognosis [29-31].

From our review, the most frequent diagnostic method for cardiac tamponade was an echocardiogram. This is a relatively inexpensive, non-invasive tool that can be done at the bedside and does not carry the risk of radiation like computerized tomography. Typical echocardiographic features suggestive of the onset of cardiac tamponade are early diastolic right ventricular (RV) collapse, right atrium and left atrium collapse, and phasic respiratory changes in the RV and left ventricle [32]. Though an echocardiogram remains the choice imaging in the diagnosis of tamponade, there are situations where a CT scan of the chest has more advantages. Patients with obesity have reduced acoustic windows making echocardiography technically difficult; in these patients, a CT scan of the chest is preferred due to its ability to acquire high-quality motion-free images of the pericardium [33]. Also, a CT scan has a larger field of view which provides an assessment of the whole chest, making it possible to detect conditions that may mimic pericardial effusion such as massive pleural effusion, pericardial masses or other mediastinal diseases [33].

Our review found that the most common diagnostic tool for the pathology of tamponade was cytology which is consistent with findings in the literature [34]. Cytology may have been done more frequently compared to biopsy because the pericardium is more difficult to biopsy compared to other mesothelial sites such as the pleura and peritoneal [35]. Furthermore, Karpathiou et al. [36] suggested that cancer cells tend to float freely in the pericardial cavity in contrast to pleural metastases, which tend to be more invasive of the pleura; hence, easier to obtain cytology of the free-floating cancer cells. More importantly, cytology has been reported to have a higher cancer detection rate of malignant pericardial effusion compared to biopsy [34]. These reasons may explain why cytology of pericardial effusion is more frequently performed resulting in more reported cases of pathologic diagnosis of pericardial effusion and tamponade with cytology than biopsy. However, a combination of both cancer detection methods (cytology and biopsy) may improve the diagnostic accuracy of the pathology of pericardial cells [37]. The presence of neoplastic cells in pericardial effusion has been found to be associated with poor prognosis [38-40].

The initial treatment of cardiac tamponade was pericardiocentesis in all the included studies in our review [1-4,7-18]; in some cases, this was enough to treat the tamponade. However, others received adjunct therapies such as surgery (pericardial window or pericardiectomy) or chemotherapy (local intra-pericardial instillation or systemic). There has been an ongoing debate as regards which treatments are most effective. Some advocate a combination of pericardiocentesis and intrapericardial instillation of sclerosing agents because they found the recurrence rate of pericardial effusion lower in their study compared to other studies where the patients were treated with pericardiocentesis alone [41]. Reynolds et al. [42] suggested that pericardiocentesis should be followed by systemic chemotherapy for malignant pericardial effusion in patients with breast cancer because this method was deemed to achieve local control of pericardial effusion and improve survival. However, according to Bishiniotis et al. [1], a combination of pericardiocentesis, local instillation of sclerosing agents and systemic adjuvant therapy achieves satisfactory survival outcomes. Some of the local intra-pericardial therapy that has been described in the literature include tetracycline, thiotepa, cisplatin, bleomycin, atabrine hydrochloride and sclerotherapy [1,2,4,9,12,14,16,38]. Other authors adopted a more aggressive approach by creating a pericardial window (pericardiectomy) in addition to pericardiocentesis and chemotherapy which they argue provides better survival [2]. Nevertheless, some authors hold the opinion that patients with malignant cardiac tamponade managed conservatively had a longer symptom-free interval than those who had surgical interventions such as the pericardial window in other studies [43].

From the aforementioned, it is clear that there is currently no agreement on the optimal adjunct therapy in the management of cardiac tamponade in breast cancer after an initial pericardiocentesis. As pointed out by Press et al. [44], due to the lack of controlled trials and large observational studies in this area, it is difficult to make definite conclusions on the optimal adjunct treatment due to different histologic types of cancer, lack of uniformity in defining clinical response, and concurrent therapy in many of these patients.

The median survival time of patients with cardiac tamponade was 13 months in our study; this seems better than what was reported in lung cancer, which is the commonest cause of cardiac tamponade [44-47]. Mortality in our review was largely unrelated to re-accumulation of pericardial effusion or recurrence of cardiac tamponade. We also did not find any significant association between time to tamponade and survival. This may suggest that other factors such as tumor burden and underlying comorbidities may play a role in the survival outcomes of these patients. More observational studies are needed to determine the predictors of survival in this population of patients.

Our study does have some limitations. Firstly, the publications in the literature on this subject comprise primarily case reports and case series, with few cohort studies. Case reports and case series are subject to the risk of selection and publication biases since positive findings tend to be more commonly published than negative ones. In addition, our review included a relatively small number of cases due to the rarity of cardiac tamponade in breast cancer. Therefore, the general application of our findings may be difficult and should be interpreted cautiously.

Despite these limitations, a systematic review of the case reports and case series, with the few observational studies, was important to summarize the available data on cardiac tamponade in patients with breast cancer to provide insight into the characteristics and survival of this specific patient population. More observational studies are needed to establish the predictors, survival, and optimal therapy of cardiac tamponade in breast cancer which will assist in making evidence-based decisions in managing such patients.

## Conclusions

Cardiac tamponade though rare, may adversely affect survival in patients with breast cancer. Early diagnosis with echocardiogram and cytology may guide management and expectations. More observational studies are needed to establish the predictors, survival, and optimal therapy of cardiac tamponade in breast cancer which will assist in making evidence-based decisions in managing such patients.

## Appendices

**Table 5:** Tool for assessing the quality and risk of bias of case reports and case series

Domains	Question items in the respective domains (weighted score in points)
Selection	Does the patient(s) represent(s) the whole experience of the investigator (center) or is the selection method unclear to the extent that other patients with a similar presentation may not have been reported? (1 point)
Ascertainment	Was the exposure adequately ascertained? (0.5 points)
	Was the outcome adequately ascertained? (0.5 points)
Causality	Was follow-up long enough for outcomes to occur? (1 point)
Reporting	Is the case(s) described with sufficient details to allow other investigators to replicate the research or to allow practitioners to make inferences related to their own practice? (1 point)

**Table 6:** Results of quality assessment

Author	Study Design	Selection	Ascertainment	Causality	Reporting	Total	Rating
Almagro, 1982 [7]	case report	0	1	0	1	2	Low
Almajed, 2022 [8]	case report	0	1	0	1	2	Low
Bishiniotis, 2000 [1]	cohort	1	1	1	1	4	Good
Bitran, 1984 [2]	cohort	1	1	1	1	4	Good
Davis, 1978 [9]	case report	0	0.5	1	0	1.5	Low
Einama, 2006 [10]	case report	0	1	1	1	3	Moderate
Ikeda, 2013 [11]	case report	0	1	1	1	3	Moderate
Imamura, 1989 [12]	case report	0	1	0	1	2	Low
Ismail, 1991 [13]	case report	0	1	0	1	2	Low
Konishi, 2012 [14]	case series	0	1	1	1	3	Moderate
Koyama, 1999 [15]	case report	0	1	0	1	2	Low
Lv, 2016 [16]	case report	0	1	1	1	3	Moderate
Ramakrishnan, 1988 [3]	cohort	1	1	1	1	4	Good
Stitt, 1987 [17]	case report	0	1	1	1	3	Moderate
Ucche, 2022 [18]	case report	0	1	1	1	3	Moderate
Woll, 1987 [4]	cohort	1	1	1	1	4	Good
